# Model-Free High-Order Sliding Mode Controller for Station-Keeping of an Autonomous Underwater Vehicle in Manipulation Task: Simulations and Experimental Validation

**DOI:** 10.3390/s22124347

**Published:** 2022-06-08

**Authors:** Josué González-García, Alfonso Gómez-Espinosa, Luis Govinda García-Valdovinos, Tomás Salgado-Jiménez, Enrique Cuan-Urquizo, Jesús Arturo Escobedo Cabello

**Affiliations:** 1 Tecnologico de Monterrey, Escuela de Ingenieria y Ciencias, Av. Epigmenio González 500, Fracc. San Pablo, Queretaro 76130, Mexico; a01208772@tec.mx (J.G.-G.); ecuanurqui@tec.mx (E.C.-U.); arturo.escobedo@tec.mx (J.A.E.C.); 2 Center for Engineering and Industrial Development (CIDESI), Energy Division, Queretaro 76125, Mexico; tsalgado@cidesi.edu.mx

**Keywords:** AUV, station-keeping, SMC, finite-time

## Abstract

The use of autonomous underwater vehicles (AUVs) has expanded in recent years to include inspection, maintenance, and repair missions. For these tasks, the vehicle must maintain its position while inspections or manipulations are performed. Some station-keeping controllers for AUVs can be found in the literature that exhibits robust performance against external disturbances. However, they are either model-based or require an observer to deal with the disturbances. Moreover, most of them have been evaluated only by numerical simulations. In this paper, the feasibility of a model-free high-order sliding mode controller for the station-keeping problem is validated. The proposed controller was evaluated through numerical simulations and experiments in a semi-Olympic swimming pool, introducing external disturbances that remained unknown to the controller. Results have shown robust performance in terms of the root mean square error (RMSE) of the vehicle position. The simulation resulted in the outstanding station-keeping of the BlueROV2 vehicle, as the tracking errors were kept to zero throughout the simulation, even in the presence of strong ocean currents. The experimental results demonstrated the robustness of the controller, which was able to maintain the RMSE in the range of 1–4 cm for the depth of the vehicle, outperforming related work, even when the disturbance was large enough to produce thruster saturation.

## 1. Introduction

Autonomous underwater vehicles (AUVs) have been widely used in recent years as an alternative to extremely costly, time-consuming, and risky human underwater operations [1]. Most of the tasks performed by these vehicles are for data-gathering applications [2]. However, there is an increasing interest for their use in inspection, maintenance, and repair operations [3] that require manipulation and interaction with objects in the underwater environment. Nowadays, this is mainly performed by remotely operated vehicles (ROVs) equipped with sensors, actuators, or manipulators designed for these specific tasks. Human intervention to remotely control the manipulator enables these missions by allowing them to respond to changes caused by the unpredictable underwater environment. Nevertheless, recent research has addressed the full automation of such tasks [4,5,6,7], including the implementation of collaborative AUVs [8,9,10,11,12].

Regardless of whether the manipulation task is performed by humans or autonomously, fulfilling some operations, such as precise navigation and station-keeping, is a challenge for researchers. These operations are quite difficult to achieve due to unknown external disturbances and the highly nonlinear hydrodynamics of the vehicle [13]. Autonomous navigation of AUVs has been widely explored in its different variants: waypoint navigation, path following, and trajectory tracking. For this purpose, various techniques, such as sliding mode control (SMC), high-order SMC, adaptive control, backstepping control, fuzzy control, and neural network control [14,15,16,17,18,19], have been explored. In manipulation operations, autonomous navigation is used to drive the vehicle to the object or surface on which the task will be performed. Once the vehicle reaches its position, it should maintain its position and orientation during the manipulation, overcoming unknown external disturbances caused either by the underwater environment or the manipulation task itself. This is known as station-keeping [20] and is critical to the success of a mission as it allows the operator or autonomous manipulator to have better control of the operation and reduces the risks of collisions that could damage either the vehicle, the manipulator, or the object.

Sakiyama et al. [21] proposed a disturbance observer for the station-keeping of AUVs. Numerical simulations were performed introducing external disturbances of 10 N in the horizontal plane. The results showed an initial push for the vehicle of no more than 5 cm, which is compensated after a fraction of a second. Ding et al. [20] investigated a modified adaptive generalized super-twisting algorithm supported by an adaptive tracking differentiator and reduced-order extended state observer for free-floating manipulation under model uncertainties and external disturbances. The authors performed numerical simulations to verify the feasibility and efficiency of their proposed control scheme when sensor noise, external disturbances, and 30% of parameter uncertainties were introduced. The RMSE for the position was kept below 0.5 cm and 0.2° for orientation. Vu et al. [22] designed an SMC-based station-keeping algorithm for an AUV subjected to model uncertainties and ocean current disturbances in the horizontal plane. In simulations, the authors used a random value between −35% and 35% as model uncertainties and included ocean currents in the *x, y,* and *z* axes with a mean value of 0.5 m/s. At the beginning of the simulations there was a shift of the AUV position with a maximum error of 5 cm. After a couple of seconds, the vehicle returns to the desired position. Experiments with the AUV with an unknown added mass and an external current of 1 knot resulted in a position drift of up to 20 cm. It is undeniable that real-world experiments are very challenging. Even control systems that show robust and exceptional performance in numerical simulations are subject to performance drift when used in the real world. A model-free high-order SMC with finite-time convergence for trajectory tracking of AUVs was proposed by González-García et al. [15,23] and validated by numerical simulations and experiments. In simulations, the proposed controller brought tracking errors to zero in a user predefined time-base. The performance of the controller was then evaluated in a semi-Olympic swimming pool where the vehicle followed depth and heading trajectories with an RMSE of 1 cm and 2.7°, respectively. The authors hypothesize that this control scheme can be used for cooperative tasks involving multiple robots, such as trajectory tracking and station-keeping.

This work aims to evaluate the feasibility of using the model-free high-order SMC with finite-time convergence for the station-keeping problem on an AUV. Numerical simulations were performed using the BlueROV2 hydrodynamic model considering three-dimensional space. Pool experiments were performed with the BlueROV2 platform in the *z*-axis. External disturbances were introduced in the simulations as strong ocean currents. In the pool experiments, the disturbances were introduced by installing an additional thruster aligned with the *z*-axis of the robot, which can generate a disturbance of up to 50 N.

## 2. Materials and Methods

This section presents the kinematics and hydrodynamics of AUVs, the BlueROV2 model used in the simulations, the hardware and software configuration of the BlueROV2 vehicle, and the setup of the experiments performed in this work.

### 2.1. Underwater Vehicles Kinematics and Hydrodynamics

**Figure 1:**
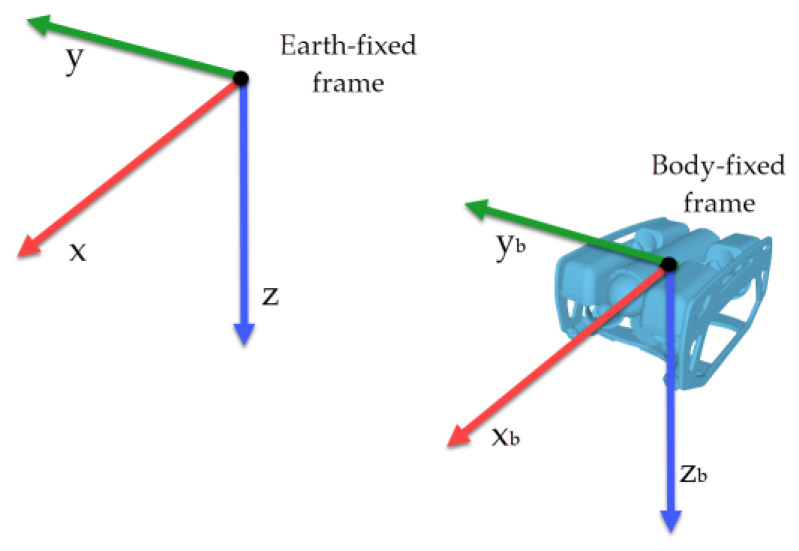
Reference frames for underwater vehicles.

The kinematics of an underwater vehicle can be described by two reference frames [24], as shown in Figure 1. The orthonormal axes are called *x*, *y*, and *z* for the Earth-fixed frame and$\;x_{b}$, $y_{b}$, and $z_{b}$ for the Body-fixed frame.

The Society of Naval Architects and Marine Engineers (SNAME) has defined a convention for notating the position, orientation, velocities, forces, and moments of an underwater vehicle, as shown in Table 1. 

The position and orientation of the vehicle in the Earth-fixed frame are described as η, its velocities in the Body-fixed frame as ν, and the forces and moments in the Body-fixed frame as τ:

(1) $$\eta ={\left(\eta_{1},\eta_{2}\right)}^{T}={\left(x,y,z,\phi ,\theta ,\psi \right)}^{T},$$

(2) $$\nu ={\left(\nu_{1},\nu_{2}\right)}^{T}={\left(u,v,w,p,q,r\right)}^{T},$$

(3) $$\tau ={\left(X,Y,Z,K,M,N\right)}^{T}.\;$$

Fossen [24] describes the hydrodynamic model of underwater vehicles by Newton–Euler Equations as

(4) $$M\dot{\nu }+C\left(\nu \right)\nu +D\left(\nu \right)\nu +g\left(\eta \right)=\tau +\omega ,$$

(5) $$\tau =B_{t}u_{t},$$

where

M∈R^6×6^ is the inertial and added mass matrix,

C∈R^6×6^ is the rigid body and added mass centripetal and Coriolis matrix,

D∈R^6×6^ is the hydrodynamic damping matrix,

g∈R^6^^×1^ is the restitution forces vector, 

$B_{t}\in R$^6×6^ is the thruster allocation matrix, 

$u_{t}\in R$^6×1^ is a vector containing the force generated by the thrusters, and

ω∈R^6×1^ represents external disturbances.

**Property** **1.** *The inertia matrix *M* is symmetric, positive definite, and constant, i.e., *$M=M^{T}>0$, $\dot{M}=0$;

**Property** **2.** *The Coriolis and centripetal matrix*$C\left(\nu \right)$*is skew-symmetric, i.e.*, $C\left(\nu \right)=-C^{T}\left(\nu \right)$, $\forall \nu \in {\mathbb{R}}^{n}$. *Thus*, $\nu^{T}C\left(\nu \right)\nu =0$, $\forall \nu \in {\mathbb{R}}^{n}$, ν≠0;

**Property** **3.** *The damping matrix*$D\left(\nu \right)$*is nonsymmetric and strictly positive, i.e.*, $D\left(\nu \right)>0$, $\forall \nu \in {\mathbb{R}}^{n}$.

**Property** **4.** *The dynamic model of an underwater vehicle is linearly parametrizable by the product of a regressor*$Y\left(\eta ,\dot{\eta },\ddot{\eta }\right)\in {\mathbb{R}}^{n\times p}$*composed of known functions and a vector*$\theta \in {\mathbb{R}}^{p}$*composed of dynamic parameters, that is*, $Y\left(\eta ,\dot{\eta },\ddot{\eta }\right)\in {\mathbb{R}}^{n\times p}\theta \in {\mathbb{R}}^{n}.$

**Property** **5.** *Boundedness of dynamic terms. For constant*$\beta_{i}>0$:

- *The inertia matrix*M*satisfies the following*:
  (6) $$\beta_{1}<\lambda_{m}\left(M\right)\leq ||M||\leq \lambda_{M}\left(M\right)<\beta_{2}\;$$
  *with*$\lambda_{m}$*and*$\lambda_{M}$*denoting the minimum and maximum eigenvalue of*M, *respectively*.
- *The Coriolis and centripetal matrix*$C\left(\nu \right)\;$*satisfies the following*:
  (7) $$\|C(\nu )\|\leq \beta_{3}||\nu ||,\forall \nu \in {\mathbb{R}}^{n}.$$
- *The damping matrix*$D\left(\nu \right)$*satisfies the following*:
  (8) $$\|D\left(\nu \right)\|\leq \beta_{4}||\nu ||,\forall \nu \in {\mathbb{R}}^{n}.\;$$
- *The vector of restoring forces*$g\left(\eta \right)\in {\mathbb{R}}^{n}$*satisfies the following*:
  (9) $$\|g\left(\eta \right)\|<\beta_{5}.\;$$

A series of kinematic transformations can be applied to Equation (4) to express the model of the vehicle in the Earth-fixed frame

(10) $$\dot{\eta }=J\left(\eta_{2}\right)\nu ↔\;\nu =J^{-1}\left(\eta_{2}\right)\dot{\eta },\;$$

(11) $$\ddot{\eta }=J\left(\eta_{2}\right)\dot{\nu }+\dot{J}\left(\eta_{2}\right)\nu ↔J^{-1}\left(\eta_{2}\right)\left[\ddot{\eta }-J\left(\eta_{2}\right)\nu \right],$$

with

(12) $$J\left(\eta_{2}\right)=\left[\begin{array}{cc} J_{1}\left(\eta_{2}\right) & 0_{3\times 3}\\ 0_{3\times 3} & J_{2}\left(\eta_{2}\right) \end{array}\right],$$

and

(13) $$J_{1}\left(\eta_{2}\right)=\left[\begin{array}{ccc} c_{\theta }c_{\psi } & -s_{\psi }c_{\phi }+s_{\phi }s_{\theta }c_{\psi } & s_{\phi }s_{\psi }+s_{\theta }c_{\phi }c_{\psi }\\ s_{\psi }c_{\phi } & c_{\phi }c_{\psi }+s_{\phi }s_{\theta }s_{\psi } & -c_{\phi }c_{\psi }+s_{\theta }s_{\psi }c_{\phi }\\ -s_{\theta } & s_{\phi }c_{\theta } & c_{\phi }c_{\theta } \end{array}\right],$$

(14) $$J_{2}\left(\eta_{2}\right)=\left[\begin{array}{ccc} 1 & s_{\phi }t_{\theta } & c_{\phi }t_{\theta }\\ 0 & c_{\phi } & -s_{\phi }\\ 0 & \frac{s_{\phi }}{c_{\theta }} & \frac{c_{\phi }}{c_{\theta }} \end{array}\right],$$

(15) $$\eta_{2}=\left[\phi ,\theta ,\psi \right],$$

where $J_{1}\left(\eta_{2}\right)\in {\mathbb{R}}^{3\times 3}$ and $J_{2}\left(\eta_{2}\right)\in {\mathbb{R}}^{3\times 3}$ are the matrices relating the linear and angular velocity components, $\nu_{1}$ and $\nu_{2}$, to the Earth-fixed frame. The symbols $c_{angle}$, $s_{angle}$, and $t_{angle}$ are abbreviations for $\cos\left(angle\right)$, $\sin\left(angle\right)$, and $\tan\left(angle\right)$, respectively.

**Assumption** **1.** *To avoid a possible singularity problem in*$J\left(\eta \right)$, *the pitch angle*θ*is bounded as*

(16) $$\left|\theta \right|<\theta_{M}<\pi /2,$$

*where* $\theta_{M}$ *stands for the upper bound of* θ *and it is a known positive constant*.

**Assumption** **2.** *The Jacobian transformation matrix*$J\left(\eta \right)$*is bounded by a known positive constant*$J_{sup}$ [25] *so that*

(17) $$sup_{\eta }||J,\left(\eta \right)||\leq J_{sup}.\;$$

**Remark** **1.** *The transformation in Equation (10) is ill-posed when*θ=±90°. *A quaternion approach might be considered to overcome this singularity. However, the vehicle is not required to operate at*θ=±90°*and it is completely stable in the pitch and roll angles*.

Then, the hydrodynamic model of the underwater vehicle can be described as follows

(18) $$M_{\eta }\left(\eta \right)\ddot{\eta }+C_{\eta }\left(\nu ,\eta \right)\dot{\eta }+D_{\eta }\left(\nu ,\eta \right)\dot{\eta }+g_{\eta }\left(\eta \right)=\tau_{\eta }$$

where

(19) $$M_{\eta }\left(\eta \right)=J^{-T}\left(\eta \right)MJ^{-1}\left(\eta \right),\;$$

(20) $$C_{\eta }\left(\nu ,\eta \right)=J^{-T}\left(\eta \right)\left[C\left(\nu \right)-MJ^{-1}\left(\eta \right)\dot{J}\left(\eta \right)\right]J^{-1}\left(\eta \right),$$

(21) $$D_{\eta }\left(\nu ,\eta \right)=J^{-T}\left(\eta \right)D\left(\nu \right)\;J^{-1}\left(\eta \right),$$

(22) $$g_{\eta }\left(\eta \right)=J^{-T}\left(\eta \right)g\left(\eta \right),$$

(23) $$\tau_{\eta }=J^{-T}\left(\eta \right)\tau .$$

### 2.2. BlueROV2

**Figure 2:**
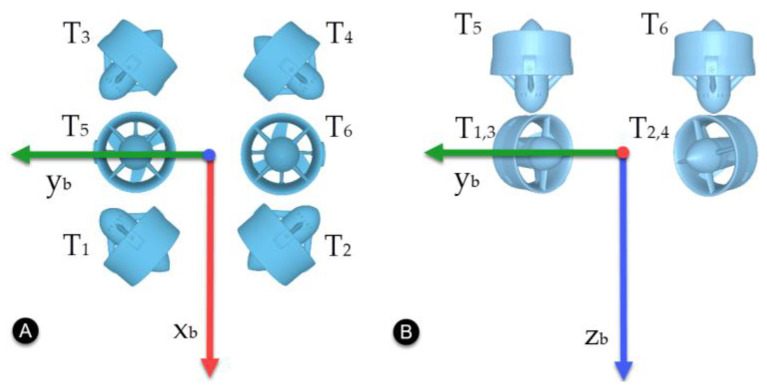
Thruster configuration for the BlueROV2. (**A**) Top view. (**B**) Front view.

The vehicle used in this work is the BlueROV2 from Blue Robotics^®^ [26]. It has a vectored configuration with six thrusters arranged as shown in Figure 2.

The thruster allocation matrix $B_{t}$ for the BlueROV2 is defined as

(24) $$B_{t}=\left[\begin{array}{cccccc} 0.7071 & 0.7071 & -0.7071 & -0.7071 & 0 & 0\\ -0.7071 & 0.7071 & -0.7071 & 0.7071 & 0 & 0\\ 0 & 0 & 0 & 0 & -1 & -1\\ 0 & 0 & 0 & 0 & 0.115 & -0.115\\ 0 & 0 & 0 & 0 & 0 & 0\\ -0.1773 & 0.1773 & -0.1773 & 0.1773 & 0 & 0 \end{array}\right].$$

**Remark** **2.** *The thruster allocation of BlueROV2 does not allows active control of the pitch angle*θ. *However, the motion around this axis is considered self-regulated due to the vehicle’s buoyant restoring moments*.

### 2.3. BlueROV2 Simulator

**Figure 3:**
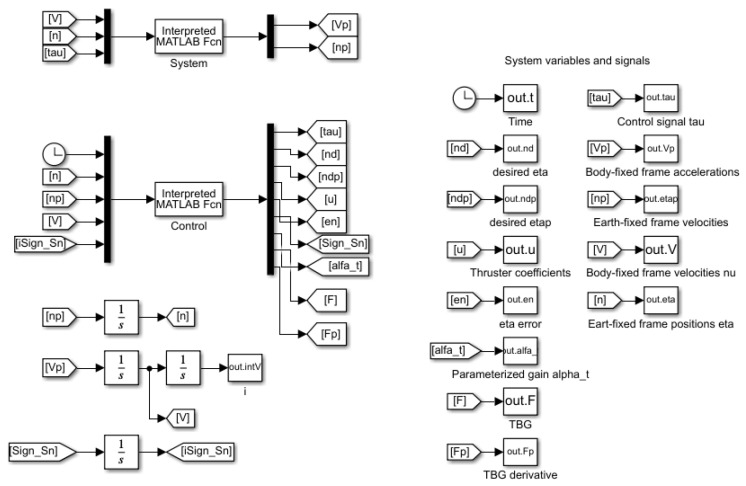
BlueROV2 simulator. Simulink block diagram.

All of the BlueROV2 hydrodynamics were compiled to create a BlueROV2 Matlab/Simulink^®^ simulator in [15]. This simulator was used to perform the numerical simulations in this work. The block diagram of the Simulink workspace is shown in Figure 3.

Ocean currents can be included as external disturbances in the simulation by using relative velocity—the difference between the real velocity and the velocity of the ocean current—as described by Fossen [24]:

(25) $$\nu_{rel}=\nu -\nu_{oc},$$

where ν is the vehicle velocity and $\nu_{oc}$ is the ocean current velocity.

A generalized vector for an irrotational ocean current velocity is described by

(26) $$\nu_{oc}={\left[u_{oc},v_{oc},w_{oc},0,0,0\right]}^{T},$$

where $u_{oc}$ is the ocean current velocity from the north, $v_{oc}$ is the ocean current velocity from the east, and $w_{oc}$ is the ocean current velocity from below.

Defining $\alpha_{oc}$ as the angle of attack and $\beta_{oc}$ as the slide slip angle, every element of the velocity vector can be calculated as

(27) $$u_{oc}=\nu_{oc}\cos\alpha_{oc}\cos\beta_{oc},\;v_{oc}=\nu_{oc}\sin\beta_{oc},\;\mathrm{and}\;w_{oc}=\nu_{oc}\sin\alpha_{oc}\cos\beta_{oc}.$$

Then, the model given by Equation (4) can be modified as follows

(28) $$M{\dot{\nu }}_{rel}+C\left(\nu_{rel}\right)\nu_{rel}+D\left(\nu_{rel}\right)\nu_{rel}+g\left(\eta \right)=\tau ,\;$$

which is the model implemented in the Matlab/Simulink^®^ simulator.

### 2.4. Experimentatal Setup

The BlueROV2 platform had its hardware and software modified so that it could be programmed to perform some underwater tasks autonomously. 

#### 2.4.1. Hardware

**Figure 4:**
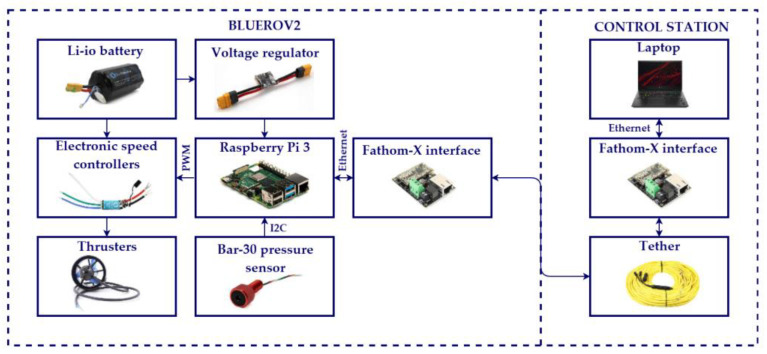
Experimental set-up, hardware configuration.

The hardware configuration used in the experiments is shown in Figure 4. A laptop with an Intel^®^ CORE i7 processor and UBUNTU 16 as the operating system was used as the control station. A tethered cable and a Fathom-X interface from BlueRobotics were used to gain remote access to the Raspberry Pi^®^ 3 (RPi) onboard the BlueROV2. This RPi is the vehicle’s processor that runs the control algorithm and manages the sensors and actuators. It runs Lubuntu as the operational system. A Bar-30 high-resolution pressure sensor by BlueRobotics was used to estimate the depth of the vehicle. It is wired to the Rpi via an I^2^C interface. The velocities of the thrusters are controlled by Pulse Width Modulation (PWM) signals sent from the RPi to a set of 30 A Electronic Speed Controllers (ESC). Finally, a 14.8 V, 18A Ah battery provides power for all the electronics. 

**Figure 5:**
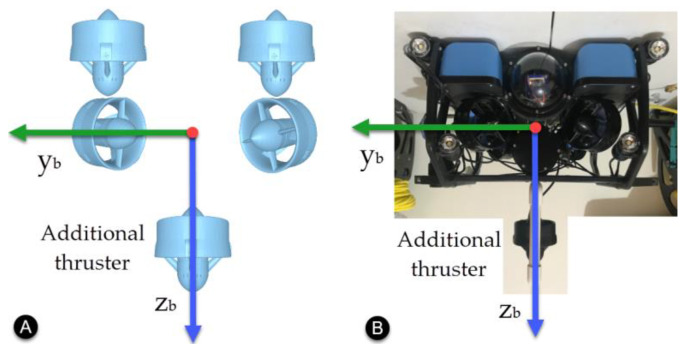
Additional thruster for external disturbance. (**A**) Front view diagram. (**B**) Implementation.

An additional thruster was added to the BlueROV2 configuration to act as an unknown external disturbance. This thruster was placed along the *z*-axis of the vehicle as shown in Figure 5.

#### 2.4.2. Software

**Figure 6:**
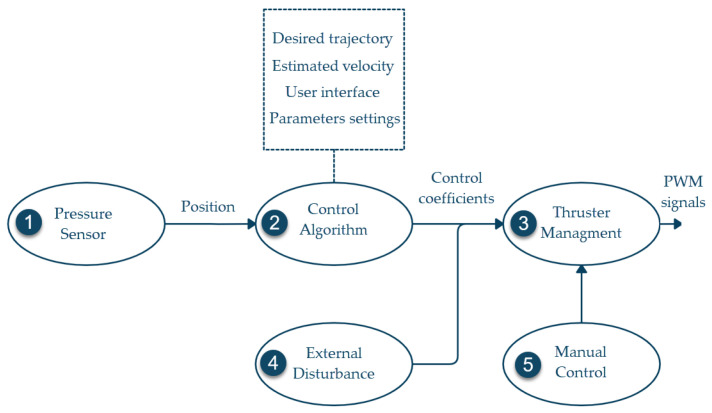
BlueROV2 software configuration.

The software was implemented using the robot operating system (ROS). The kinetic version of ROS was installed on the RPi and five nodes were programmed in Python to manage the system. A pressure sensor (1) node manages the Bar-30 sensor and estimates the depth of the robot. Then it publishes the z position of the vehicle at a 100 Hz rate. A control algorithm (2) node reads the z position, runs an exact differentiator to estimate the velocity $\dot{z}$, contains the controller parameters, executes the control algorithm, and provides a simple user interface. This node works at a 100 Hz frequency and publishes the thruster’s coefficient vector u to a thruster management (3) node, which generates the PWM signals to control the thrusters. The external disturbance (4) node introduces the unknown external disturbance into the system. It does not share the magnitude or direction of the external disturbance with the controller. Finally, a manual control (5) node was programmed to manually move the vehicle to its initial position. This software configuration is shown in Figure 6.

**Figure 7:**
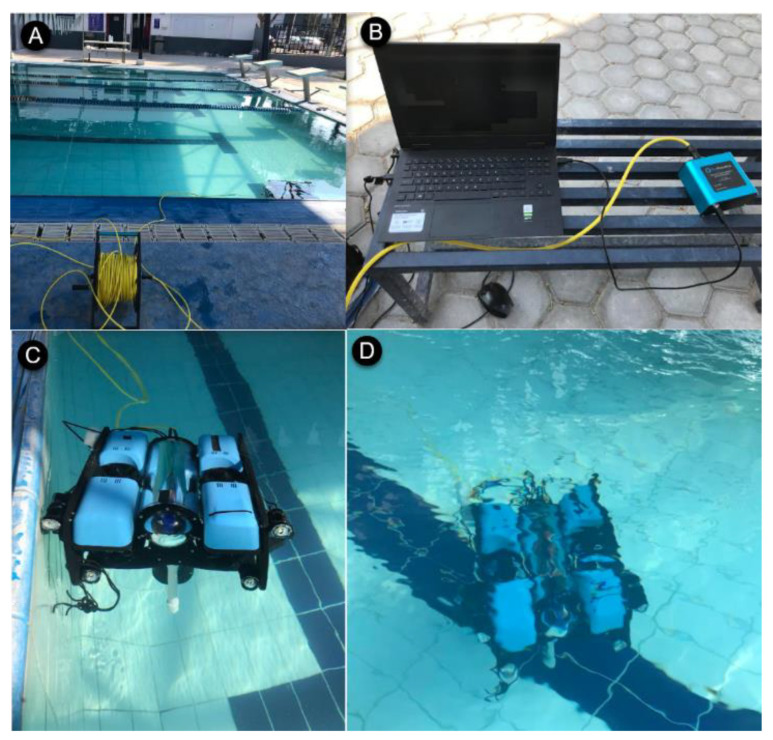
Experimental set-up. (**A**) Semi-Olympic swimming pool. (**B**) Control station. (**C**) BlueROV2 deployment. (**D**) Station-keeping task execution.

Experiments were performed in a semi-Olympic swimming pool at the Tecnologico de Monterrey, Campus Queretaro. The location and experimental setup are shown in Figure 7.

## 3. Controller Design

### 3.1. Model-Free High Order Sliding Mode Controller

The controller used in this work is a model-free high-order SMC with finite-time convergence in a predefined time [15]. According to Property 4, the Equation (18) is linearly parameterizable by the product of a regressor $Y\left(\eta ,\dot{\eta },\ddot{\eta }\right)\in {\mathbb{R}}^{n\times p}$, consisting of known nonlinear functions and a vector $\theta \in {\mathbb{R}}^{p}\;$with constant parameters. This parametrization can be rewritten in terms of a nominal reference ${\dot{\eta }}_{r}$ and its time derivative ${\ddot{\eta }}_{r}$ as

(29) $$M_{\eta }\left(\eta \right){\ddot{\eta }}_{r}+C_{\eta }\left(\nu ,\eta \right){\dot{\eta }}_{r}+D_{\eta }\left(\nu ,\eta \right){\dot{\eta }}_{r}+g_{\eta }\left(\eta \right)=Y\left(\eta ,\dot{\eta },{\dot{\eta }}_{r},{\ddot{\eta }}_{r}\right)\theta .\;$$

Subtracting Equation (29) from both sides of Equation (18) results in the open-loop error hydrodynamics expression:

(30) $$M_{\eta }\left(\eta \right){\dot{S}}_{r}+C_{\eta }\left(\nu ,\eta \right)S_{r}+D_{\eta }\left(\nu ,\eta \right)S_{r}=\tau_{\eta }-Y\left(\eta ,\dot{\eta },{\dot{\eta }}_{r},{\ddot{\eta }}_{r}\right)\theta ,$$

where $S_{r}=\dot{\eta }-{\dot{\eta }}_{r}$ is called the extended error. 

The nominal reference ${\dot{\eta }}_{r}$ is defined in terms of the position and velocity paths as follows

(31) $${\dot{\eta }}_{r}={\dot{\eta }}_{d}-\alpha \tilde{\eta }+S_{d}-K_{i}\int_{0}^{t}sign\left(S_{\eta }\right)d\sigma ,$$

where $\tilde{\eta }=\eta -\eta_{d}$ is the tracking error of the position, $\eta_{d}$ is the desired trajectory, $K_{i}$ is a diagonal positive definite n×n gain matrix, α is a gain yet to be defined, $sign\left(x\right)$ is the signum function of the vector x, and

(32) $$S=\dot{\tilde{\eta }}+\alpha \tilde{\eta }\;,$$

(33) $$S_{d}=S\left(t_{0}\right)e^{-kt},$$

(34) $$S_{\eta }=S-S_{d},$$

with k>0.

**Assumption** **3.** *The nominal reference*${\dot{\eta }}_{r}$*and its derivative*${\ddot{\eta }}_{r}$*are bounded by positive scalars*$\beta_{i},\;i=6,\ldots ,9$*as follows*:

(35) $$||{\dot{\eta }}_{r}||\;\leq \;\beta_{6}\;+\;||\alpha ||\;||\tilde{\eta }||\;+\;\lambda_{M}\left(K_{i}\right)I<\beta_{7},\;||{\ddot{\eta }}_{r}||\;\leq \;\beta_{6}\;+\;||\alpha ||\;||\dot{\tilde{\eta }}||\;<\;\beta_{9}.$$

The extended error $S_{r}$ can be rewritten as

(36) $$S_{r}=S_{\eta }+K_{i}\;\int_{0}^{t}sign\left(S_{\eta }\right)d\sigma \;,$$

and its derivative as

(37) $${\dot{S}}_{r}={\dot{S}}_{\eta }+sign\left(S_{\eta }\right),$$

from which the model-free high-order SMC is obtained with the following control law:

(38) $$\tau_{\eta }=-K_{d}S_{r}\;,$$

where $K_{d}$ is a diagonal definite positive n×n gain matrix.

**Remark** **3.** *Note that the controller does not requires any knowledge of the hydrodynamics or parameters of the vehicle.*

### 3.2. Time Parametrization of α Gain

When the α gain in Equation (32) is set to a constant value, the control law given in Equation (38) leads to an 2nd order SMC with asymptotic convergence, as reported in [27]. The controller proposed for this work achieves finite-time convergence by replacing α with a time-varying gain $\alpha \left(t\right)$. Simulation and experimental results for this controller have been reported in [15,23]. According to Parra-Vega [28], the $\alpha \left(t\right)\;$gain can be defined as:

(39) $$\alpha \left(t\right)=\left\{\begin{array}{c} \alpha_{0}\frac{\dot{\xi }\left(t\right)}{\left(1-\xi \left(t\right)\right)+\delta },\;0\leq t\leq t_{b}\\ \alpha_{c},\;t>t_{b} \end{array}\right.,$$

where $\alpha_{0}=1+\varepsilon ,\;0<\varepsilon \ll 1,$ 0<δ≪1, and $\alpha_{c}$ > 0.

A time base generator (TBG) $\xi \left(t\right)\;$is used to provide a smooth transition from 0 to 1, the duration of which can be controlled by the user with a time-base parameter $\left(t_{b}\right)$. This TBG is given by

(40) $$\xi \left(t\right)=10\;\frac{{\left(t-t_{0}\right)}^{3}}{{\left(t_{b}-t_{0}\right)}^{3}}-15\frac{{\left(t-t_{0}\right)}^{4}}{{\left(t_{b}-t_{0}\right)}^{4}}+6\frac{{\left(t-t_{0}\right)}^{5}}{{\left(t_{b}-t_{0}\right)}^{5}}\;,$$

and its derivative

(41) $$\dot{\xi }\left(t\right)=30\;\frac{{\left(t-t_{0}\right)}^{2}}{{\left(t_{b}-t_{0}\right)}^{3}}-60\frac{{\left(t-t_{0}\right)}^{3}}{{\left(t_{b}-t_{0}\right)}^{4}}+30\frac{{\left(t-t_{0}\right)}^{4}}{{\left(t_{b}-t_{0}\right)}^{5}},$$

gives a bell-shaped velocity profile.

For Equations (40) and (41), the following conditions hold: $\xi \left(t_{b}\right)=1,\;\xi \left(t_{0}\right)=\dot{\xi }\left(t_{0}\right)=\dot{\xi }\left(t_{b}\right)=0$, where $t_{0}$ represents the initial time.

**Remark** **4.** *The time base parameter*$t_{b}$*can be arbitrarily chosen by the user and does not depend on the initial conditions, parameters, or hydrodynamics of the vehicle*.

Finally, the solution of the differential equation in Equation (32) is

(42) $$\tilde{\eta }\left(t\right)=\tilde{\eta }\left(t_{0}\right){\left[1-\xi \left(t\right)+\delta \right]}^{\alpha_{0}}.\;$$

Equation (42) represents a family of solutions converging smoothly to a small value. Since $\xi \left(t_{b}\right)=1$ when $t=t_{b}$, the solution becomes

(43) $$\tilde{\eta }\left(t_{b}\right)=\tilde{\eta }\left(t_{0}\right)\delta^{\alpha_{0}},$$

when the time base is reached.

### 3.3. Stability Analysis

**Theorem** **1.** *In a closed-loop system, the control law described by Equation (38) and the model described by Equation (30) lead to*

(44) $$M_{\eta }\left(\eta \right){\dot{S}}_{r}=-K_{d}S_{r}-Y\left(\eta ,\dot{\eta },{\dot{\eta }}_{r},{\ddot{\eta }}_{r}\right)\theta -C_{\eta }\left(\nu ,\eta \right)S_{r}-D_{\eta }\left(\nu ,\eta \right)S_{r},$$

*where finite-time tracking is guaranteed if* $\;K_{d}\;$ *and* $\;K_{i}\;$ *are large enough for small initial error conditions.*

**Proof** **of** **Theorem 1.** The stability analysis has been divided into two parts:

Part I proves the stability of tracking errors with all the closed-loop signals bounded.

Part II proves that the velocity and position tracking errors converge to zero.

Part I. Boundedness of the closed loop trajectories.

Consider the following Lyapunov candidate function

(45) $$V=\frac{1}{2}S_{r}^{T}M_{\eta }\left(\eta \right)S_{r},$$

and its time derivative

(46) $$\dot{V}=-S_{r}^{T}K_{d}S_{r}-S_{r}^{T}D_{\eta }\left(\nu ,\eta \right)S_{r}-Y\left(\eta ,\dot{\eta },{\dot{\eta }}_{r},{\ddot{\eta }}_{r}\right)\theta ,$$

where the skew-symmetric property described in Property 2 was applied and the norm of $Y\left(\eta ,\dot{\eta },{\dot{\eta }}_{r},{\ddot{\eta }}_{r}\right)\theta$ was replaced by an upper bound defined by a state-dependent function $\rho \left(t\right)$.

According to Property 5 and Assumption 3, the desired trajectories and vehicle dynamics are bounded—there are upper bounds for $M_{\eta },C_{\eta },D_{\eta },g_{\eta },{\dot{\eta }}_{r},{\ddot{\eta }}_{r}$—so it can be proven that $Y\left(\eta ,\dot{\eta },{\dot{\eta }}_{r},{\ddot{\eta }}_{r}\right)\theta \;$is also upper bounded [29]. If the initial error is small enough and $K_{d}$ is large enough, one can infer the negative definiteness of outside of the small sphere $\varepsilon_{0}=\left\{S_{r}\left|\dot{V}\geq 0\right.\right\}$ centered at the origin $\dot{V}\left(S_{r}\right)=0$. This boundedness in the $L_{\infty }$ sense leads to the existence of the constant $\varepsilon_{1}>0$, so that

(47) $$||{\dot{S}}_{r}||\leq \varepsilon_{1}.\;$$

At this point, the stability of the tracking errors has been proven.

Part II. Existence of the second order sliding mode.

Consider the second order dynamical system defined by the time derivative of the nominal reference described in Equation (37) as

(48) $${\dot{S}}_{\eta }=-K_{i}sign\left(S_{\eta }\right)+S_{r}.$$

Now, consider the multiplication of Equation (48) by $S_{\eta }^{T}$:

(49) $$S_{\eta }^{T}{\dot{S}}_{\eta }=-K_{i}S_{\eta }^{T}sign\left(S_{\eta }\right)+S_{\eta }^{T}S_{r},$$

Apply Equation (47) to Equation (49), and considering $\mu =\lambda_{m}\left(K_{i}\right)-\varepsilon_{1}$ results in

(50) $$S_{\eta }^{T}{\dot{S}}_{\eta }\leq -\lambda_{m}\left(K_{i}\right)\left|S_{\eta }^{T}\left|+\right|S_{\eta }^{T}\left|||{\dot{S}}_{r}\right|\right|,\;\leq \left|S_{\eta }^{T}\right|\left(-\lambda_{m}\left(K_{i}\right)+\varepsilon_{1}\right),\;\leq -\mu \left|S_{\eta }^{T}\right|.$$

If $\lambda_{m}\left(K_{i}\right)>\varepsilon_{1}$, then μ>0. This guarantees the sliding mode at $S_{\eta }=0$ and $t_{g}=\frac{S_{\eta }\left(t_{0}\right)}{\mu }$. Note that $t_{g}=0$ for any initial condition $S_{\eta }\left(t_{0}\right)$, implying the enforcement of the sliding mode at any time and

(51) $$S_{\eta }=\dot{\tilde{\eta }}+\alpha \tilde{\eta }=0↔\;\dot{\tilde{\eta }}=-\alpha \tilde{\eta }.$$

Equation (51) implies the convergence of the tracking errors to a very small sphere centered in the origin

(52) $$(\tilde{\eta },\dot{\tilde{\eta }})=0\;$$

in a finite-time $\left(t_{b}\right)$, as described in Equations (42) and (43). □

**Remark** **5.** *Considering the solution in Equation (43) for*$t=t_{b}$, *an*$\alpha_{0}\;$*parameter very close to 1 and a very small*δ, *the tracking errors are limited to a very small neighborhood*, $\varepsilon_{2}$, *from the origin. In practice, this may represent required accuracy or a practical zero error. For*$t>t_{b},$*the time-varying gain*$\alpha \left(t\right)$*must be reset to a constant value*$\;\alpha_{c}>0$. *Since a sliding mode is induced at any time*, $\tilde{\eta }\left(t\right)\in \varepsilon_{2},\;\forall \;t>t_{b}$, *and*$\dot{\tilde{\eta }}=-\alpha_{c}\tilde{\eta }\left(t\right),\;\forall \;t>t_{b},$ $\tilde{\eta }\left(t\right)$*converges exponentially, which means that the tracking errors quickly tend to zero, leading to*

(53) $$\eta \to \eta_{d},\dot{\eta }\to {\dot{\eta }}_{d},\forall \;t>t_{b}.\;$$

**Remark** **6.** *A simple method for tuning*$\alpha_{0}$*and*δ*is to fix one of them in Equation (43). Given the initial condition*$\tilde{\eta }\left(t_{0}\right),\;$*the practical zero error*$\tilde{\eta }\left(t_{b}\right)$, *and fixing*δ, $\alpha_{0}$*is calculated as follows*:

(54) $$\alpha_{0}=\ln\left(\frac{\tilde{\eta }\left(t_{0}\right)}{\tilde{\eta }\left(t_{b}\right)}\right)\left(\frac{1}{ln\delta }\right).$$

*Fixing*$\alpha_{0}$, δ*is calculated as follows*:

(55) $$\delta =e^{\left(\frac{1}{\alpha_{0}}\right)}\ln\left(\frac{\tilde{\eta }\left(t_{0}\right)}{\tilde{\eta }\left(t_{b}\right)}\right).$$

### 3.4. Further Considerations

#### 3.4.1. Reference Frame Transformation

The control signal $\tau_{\eta }$ must be transformed from the Earth-fixed frame to the Body-fixed frame to obtain the forces and moments needed in the vehicle. This is achieved by the following transformation:

(56) $$\tau =J^{-1}\left(\eta_{2}\right)\tau_{\eta }.\;$$

Then the coefficient vector u of the thrusters is calculated as

(57) $$u=B_{t}{}^{-1}K_{T}{}^{-1}\tau ,$$

where $K_{T}$ is a diagonal matrix containing the maximum force that each thruster can deliver.

#### 3.4.2. Exact Differentiator

The tracking error $\dot{\tilde{\eta }}$ of the vehicle’s velocity is needed to calculate the control law defined in Equation (38). The sensors of BlueROV2 do not measure velocity, so the velocities of the vehicle must be estimated. A simple Euler differentiator can be used for this purpose. However, this differentiator is sensitive to noise and results in an inaccurate estimation of the vehicle velocities. This problem was addressed by programming an exact differentiator algorithm. This algorithm is based on the designed by Levant [30] and is given by:

(58) $${\dot{j}}_{0}=w_{0},$$

(59) $$w_{0}=-\lambda_{1}{\left[j_{0}-f\left(t\right)\right]}^{\frac{1}{2}}\;sign\left(j_{0}-f\left(t\right)\right)+j_{1},$$

(60) $${\dot{j}}_{1}=-\lambda_{2}\;sign\left(j_{0}-f\left(t\right)\right),$$

where $f\left(t\right)$ is the original signal to be differentiated, $sign\left(x\right)$ is the signum function for the argument x, and $\lambda_{1}=1.5$ $\lambda_{2}=1.1$ are constant gains. After a brief adjustment time, $j_{0}$ is considered as the original filtered signal and $j_{1}$ as its derivative.

(61) $$j_{0}=f\left(t\right),$$

(62) $$j_{1}=\dot{f}\left(t\right),$$

**Remark** **7.** *The accuracy of the exact differentiator and its sensitivity to noise degrades as a function of an increase of its parameters and sampling time. However, the performance drop is expected for high-order differentiations (refer to* [30]*). Since the differentiator in this work considers only the first derivative, sensitivity to noise is not considered a problem.*

**Figure 8:**
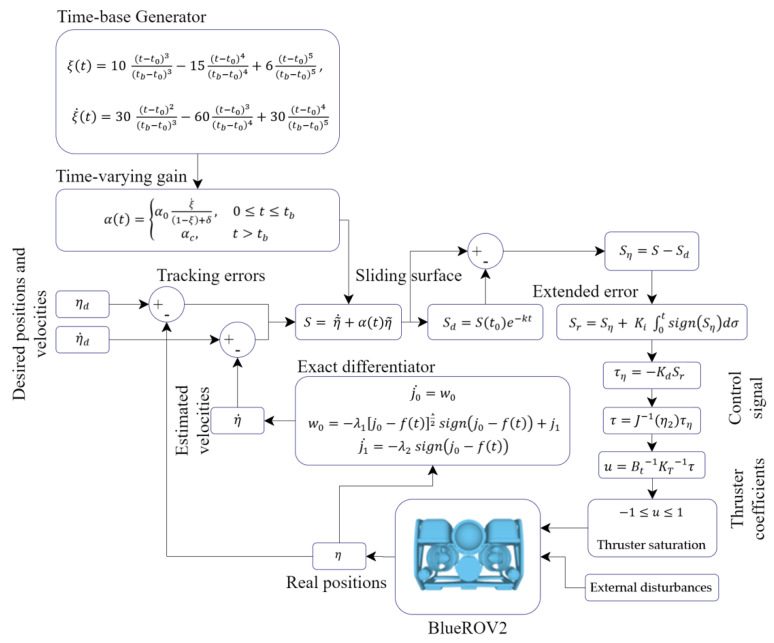
Block diagram of the proposed controller.

The complete control scheme is shown in the block diagram in Figure 8.

## 4. Results and Discussion

This section contains the results of the numerical simulations and the experiments performed for the station-keeping problem. In both scenarios, there are two test phases: autonomous navigation to the desired position and the station-keeping for the BlueROV2 position. Since the performance and robustness of the controller have been evaluated in previous work for the trajectory tracking problem, the discussion in this paper focuses on the station-keeping phase of the tests.

### 4.1. Numerical Simulations

For an initial validation of the ability model-free high-order SMC with finite-time convergence in a predefined time to achieve station-keeping, numerical simulations were performed. The controller parameters used in this work are the same as those used in the validation of the trajectory tracking problem in [15]. An initial set of parameters was established according to Section 3.3. Then simulations were performed to optimize them until the best results were obtained. The controller parameters are listed in Table 2. The sampling time for these simulations was set as variable with a maximum value of 0.01 s.

**Figure 9:**
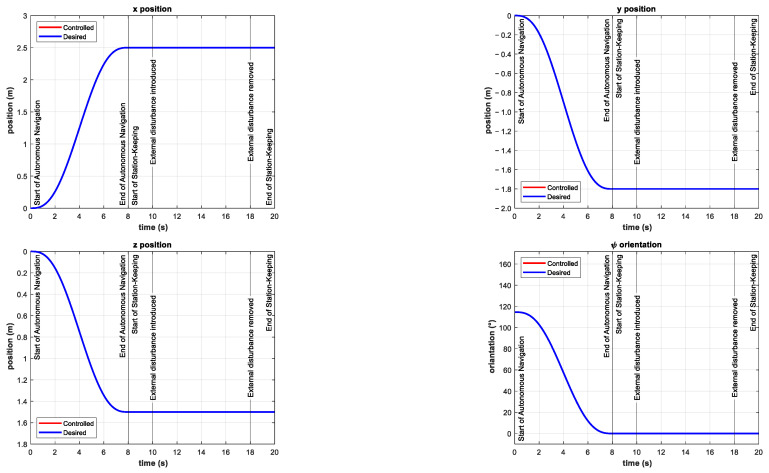
Simulation results for finite-time trajectory tracking and station-keeping in the x*,* y, z positions and ψ orientation. External disturbances were introduced in the interval 10 s≤t≤18 s as ocean currents $\left(\nu_{oc}\right)$.

The simulations consisted of the autonomous navigation of the BlueROV2 to reach the desired $\eta_{d}$ position in a predefined time-base, arbitrarily set to $t_{b}=8\;s$. After the vehicle is in the reference, the station-keeping phase of the simulation begins. In the station-keeping phase, the external disturbances $u_{oc}=0.75\frac{m}{s}$, $v_{oc}=0.25\frac{m}{s}\;$, and $w_{oc}=0.25\frac{m}{s}\;$were introduced at t=10 s as ocean currents. Then they were removed at t=18 s. The simulation results are shown in Figure 9 for the x*,* y, z positions and ψ orientation.

It can be observed that the controller is able to keep the BlueROV2 in reference for all positions and orientations. Contrary to the simulation results reported in [20,21,22], there is no initial push on the positions when the disturbance is introduced and the tracking error remains zero for all simulations. This demonstrates the robustness of the controller to overcome external disturbances quickly and effectively. 

### 4.2. Experimentation

The second validation of the proposed controller consisted of a series of experiments conducted in a semi-Olympic swimming pool. The controller parameters used in these experiments were the same as those used in the experimental validation for the trajectory tracking problem in [23]. The goal is to demonstrate that the controller can maintain the z position of the vehicle in the presence of unknown external disturbances without having to adjust its parameters in any way. The parameters for the depth control are defined in Table 3. The sampling time was set at 0.01 s.

**Figure 10:**
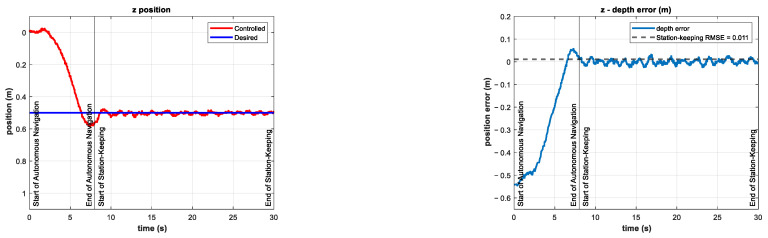
Depth (**left**) and tracking error (**right**) results of the control test. No external disturbances were introduced.

The experiments consisted of the autonomous navigation of the BlueROV2 to reach the desired z position in a predefined time base, arbitrarily set to $t_{b}=8\;s$. After the vehicle was in the reference, the station-keeping phase of the experiments began. To compare the performance of the controller in the station-keeping phase, an experiment was conducted in which no external disturbances were introduced. This experiment is referred to as the control test. The depth position and tracking error results of the control test are shown in Figure 10.

**Figure 11:**
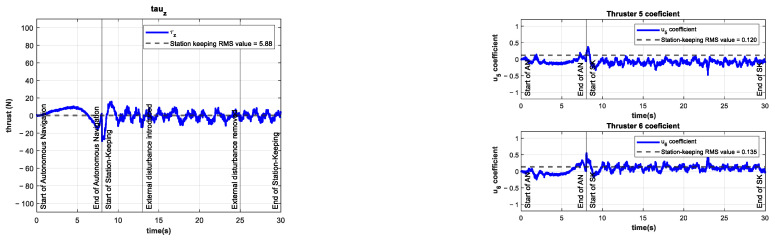
Experimental results of the control test for depth station-keeping. Control signal $\tau_{z}$ (**left**) and thruster coefficients $u_{5}$,$\;u_{6}$ (**right**). No external disturbances were introduced.

The control signal $\tau_{z}$ and the coefficients of the vertical thrusters $u_{5}$ and $u_{6}$ are shown in Figure 11.

**Figure 12:**
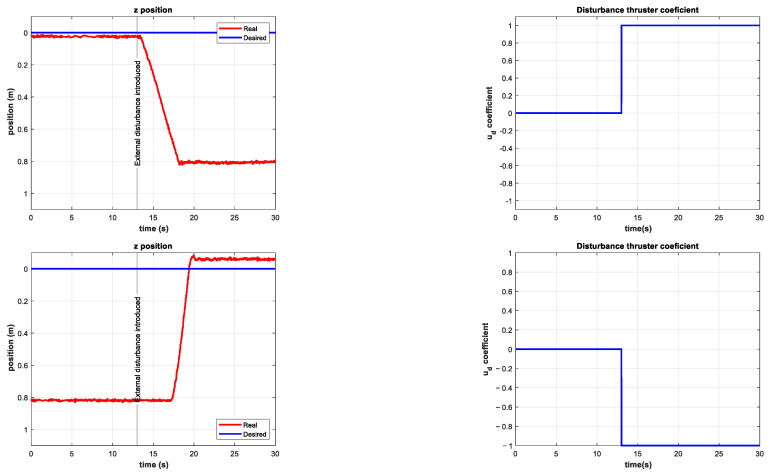
Results of the open-loop experiments. Depth of the vehicle (**upper left**) with an external disturbance of ~50 N (**upper right**) introduced at t=13 s. Depth of the vehicle (**lower left**) with an external disturbance of ~−40 N (**lower right**) introduced at t=13 s.

In the following experiments, an external disturbance—unknown to the controller—was introduced by setting the coefficient of the additional thruster to various values from −1 to +1, with a corresponding force along the *z*-axis in the range from −40 to +50 N. Some open-loop station-keeping tests were performed to demonstrate the effects of the proposed disturbance. The results of these tests are shown in Figure 12. The disturbance caused by the additional thruster drives the BlueROV2 to the bottom or top of the pool in less than 5 s.

**Figure 13:**
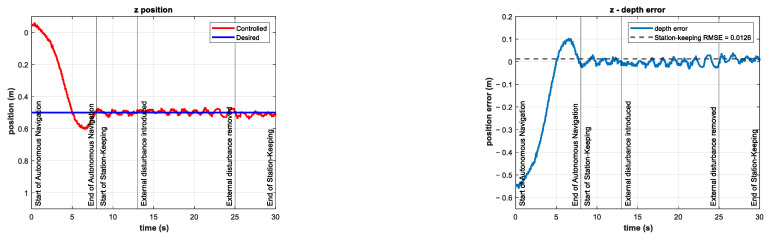
Experimental results for depth station-keeping with an external disturbance of ~10 N introduced in the interval 13 s≤t≤25 s. Depth (**left**) and tracking error (**right**).

A series of experiments were performed for station-keeping in the depth of the BlueROV2. The results for the z position and tracking error of the experiment with an external disturbance of about −10 N (with an additional thruster coefficient of −0.25) are shown in Figure 13. The resulting RMSE was 1.26 cm, which is quite similar to the RMSE from the control test. This means that the controller can maintain its performance in the presence of unknown external disturbances of the same magnitude as in the simulations in related work [21].

**Figure 14:**
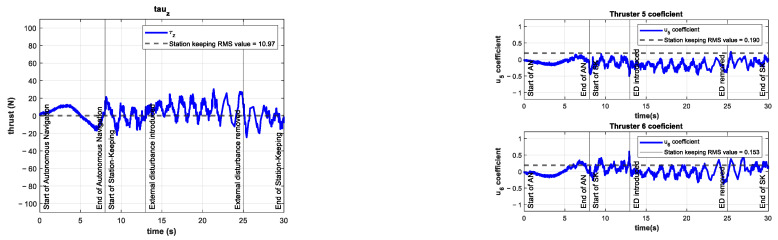
Experimental results for depth station-keeping with an external disturbance of ~10 N introduced in the interval 13 s≤t≤25 s. Control signal $\tau_{z}$ (**left**) and thruster coefficients $u_{5}$,$\;u_{6}$ (**right**).

The control signal $\tau_{z}$ and the coefficients of the vertical thrusters $u_{5}$ and $u_{6}$ are shown in Figure 14. The control signal increased its mean value by 86% compared to the control test. This higher demand on the controller is evidence of the significance of the introduced external disturbance.

**Figure 15:**
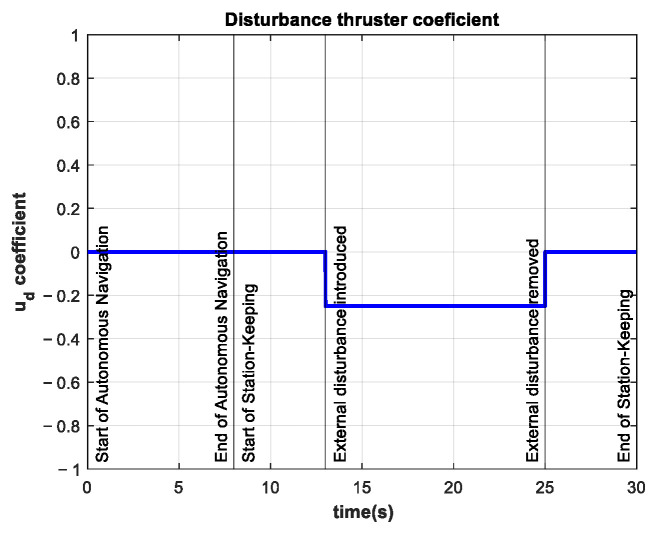
Additional thruster coefficient of −0.25 in the interval 13 s≤t≤25 s.

The coefficient for the additional thruster is shown in Figure 15. The disturbance is introduced at t=13 s and maintained until t=25 s.

**Figure 16:**
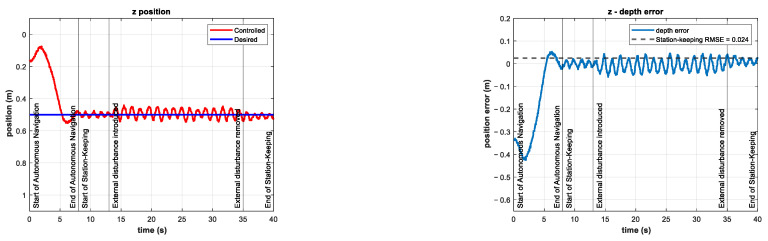
Experimental results for depth station-keeping with an external disturbance of ~20 N introduced in the interval 13 s≤t≤25 s. Depth (**left**) and tracking error (**right**).

The results for the z position and tracking error of the experiment with an external disturbance of about −20 N (with an external thruster coefficient of −0.50) are shown in Figure 16. Note that this external disturbance force is twice that of the simulations in [21]. The tracking error increases during the period when the external disturbance is introduced. The RMSE is 2.4 cm for the station-keeping phase of the experiment, which is twice as high as the RMSE of the control test. Nevertheless, this indicator is still low compared to the experimental results reported in [22].

**Figure 17:**
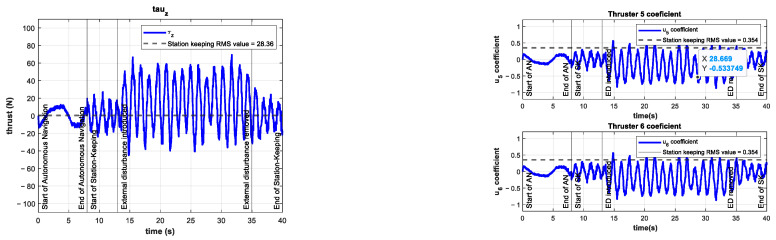
Experimental results for depth station-keeping with an external disturbance of ~20 N introduced in the interval 13 s≤t≤25 s. Control signal $\tau_{z}$ (**left**) and thruster coefficients $u_{5}$,$u_{6}$ (**right**).

As can be seen in Figure 17, the control signal $\tau_{z}$ and thus the coefficients $u_{5}$ and $u_{6}$ increase their mean value. This is due to the controller requesting more power from the thrusters in order to overcome the unknown external disturbance trying to push the vehicle out of its reference.

**Figure 18:**
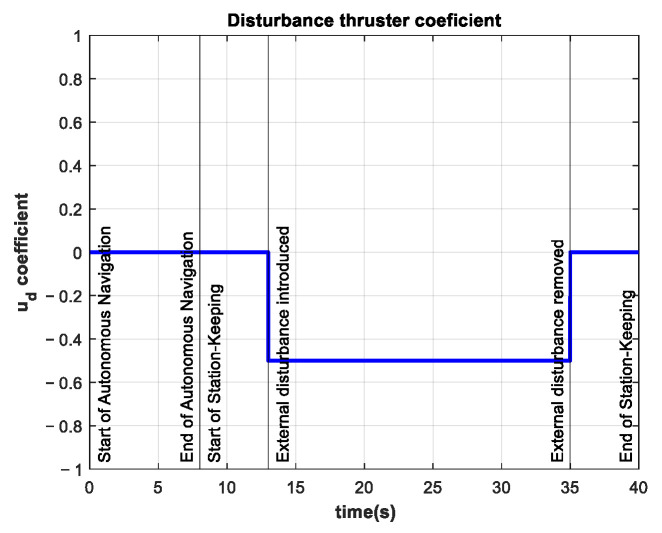
Additional thruster coefficient of −0.50 in the interval 13 s≤t≤25 s.

The control signal in this experiment is almost five times larger than the same signal in the control test. The thruster coefficients are almost three times as large as the thruster coefficients in the control test. The coefficient of the additional thruster for this experiment is shown in Figure 18.

**Figure 19:**
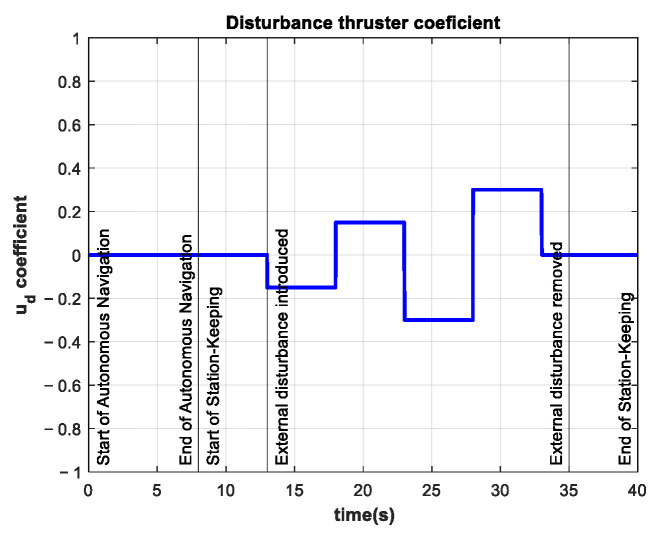
Time-varying additional thruster coefficient in the interval 13 s≤t≤25 s. Minimum value −0.25 and maximum value 0.25.

Some experiments were performed with time-varying external disturbances, such as the one shown in Figure 19. 

**Figure 20:**
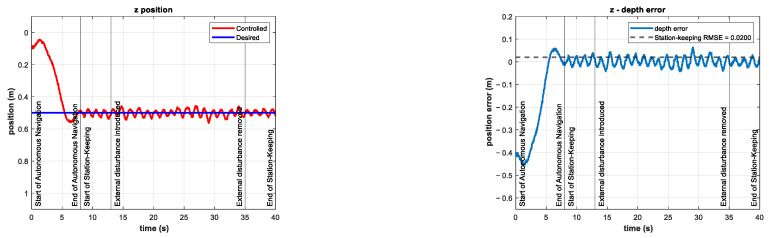
Experimental results for depth station-keeping with a time-varying external disturbance introduced in the interval 13 s≤t≤25 s. Depth (**left**) and tracking error (**right**).

**Figure 21:**
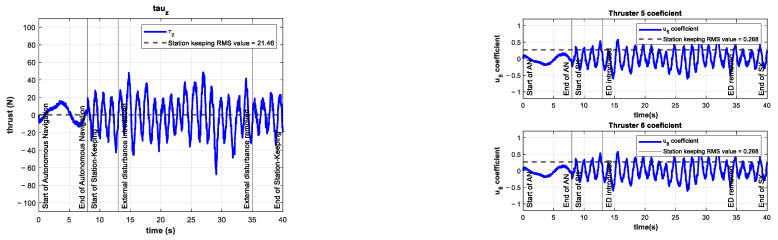
Experimental results for depth station-keeping with a time-varying external disturbance introduced in the interval 13 s≤t≤25 s. Control signal $\tau_{z}$ (**left**) and thruster coefficients $u_{5}$,$\;u_{6}$ (**right**).

In these experiments, the external disturbance changed abruptly in magnitude and direction. However, these changes did not significantly affect the performance of the controller, as shown in Figure 20 and Figure 21.

Eighteen experiments were performed, including the ones mentioned earlier, and compared with the control test. Results for the thrusters’ coefficient and depth RMSE are summarized in Table 4. 

As can be seen, the coefficients of the vertical thrusters increase when the magnitude of the external disturbance increases. The demand on the BlueROV2 vertical thrusters has increased up to 461% compared to the control test, but the controller keeps the vehicle at a small distance from the reference. The RMSE for depth station-keeping slightly increases. The maximum RMSE is 2.7 cm when the vertical thrusters do not reach saturation and 4.3 cm when they do. The results for the control signal $\tau_{z}$ are summarized in Table 5.

A comparison with the control signal in the control test is made in the third column (% vs. C-T). There, an increase of up to 853% in the energy demanded by the controller can be observed. This shows that the BlueROV2 is strongly demanded by the controller to overcome the unknown external disturbance, which it succeeds at.

## 5. Conclusions

It is critical to have a controller that can hold an AUV in position while performing manipulation tasks. In this work, a model-free high-order SMC with finite-time convergence in a predefined time, which has already been evaluated and validated for trajectory tracking, was evaluated for the station-keeping of the BlueROV2 AUV in the presence of unknown external disturbances. The external disturbance, in a range of about −40 to +50 N, was directly introduced in the *z*-axis of the BlueROV2. This external disturbance remained unknown to the controller, and the weight of the additional thruster also acts as an unknown disturbance. The controller maintained the same set of parameters and gains it had when evaluated for trajectory tracking to prove that there was no need for tuning to achieve a robust performance. Other controllers found in the literature are either model-based, require an observer for the external disturbances, need to adjust their gains online, etc., or do not perform as well as the controller proposed in this work in terms of position RMSE. Simulation results showed that the tracking error for the vehicle’s position was maintained at zero throughout the station-keeping task. The position of the vehicle did not change when an external disturbance was introduced, as was the case with other station-keeping controllers reported in the literature. Eighteen experiments were conducted in a semi-Olympic swimming pool to evaluate the proposed controller. The results have shown the robustness of the controller in terms of RMSE, which was less than 2.7 cm in all cases where there was no saturation in the vertical thrusters. Even in cases where the external disturbance abruptly changed its magnitude and direction. This is an exceptional result compared with the up to 20 cm RMSE reported in a related work where experiments were also performed with an AUV. In the cases where the external disturbance was too large and caused the saturation of the vertical thrusters, the RMSE was kept below 4.5 cm. Future work will consist of adding a sensor to the BlueROV2 to extend the experiments to the *x* and *y* axes. The goal is to program a pair of BlueROV2 AUVs with the proposed controller to perform a coordinated manipulation task.

## Figures and Tables

**Table 1:** SNAME notation for underwater vehicles.

Movement	Name	Position	Velocity	Force/Moment
*X* translation	Surge	x	u	X
*Y* translation	Sway	y	v	Y
*Z* translation	Heave	z	w	Z
*X* rotation	Roll	ϕ	p	K
*Y* rotation	Pitch	θ	q	M
*Z* rotation	Yaw	ψ	r	N

**Table 2:** Controller parameter set.

Parameter	Value	Parameter	Value
$t_{0}$	0	δ	0.001
$t_{b}$	8	k	5
$\alpha_{0}$	1.01	$k_{i}$	$diag\left[0,5,5,5,5,5\right]$
$\alpha_{c}$	20	$k_{d}$	$diag\left[800,800,800,800,0,800\right]$

**Table 3:** Controller parameters for experimentation.

Parameter	Value	Parameter	Value
$t_{0}$	0	δ	0.001
$t_{b}$	8	k	5
$\alpha_{0}$	1.005	$k_{i}$	0.05
$\alpha_{c}$	10	$k_{d}$	100

**Table 4:** Experimental results for the vertical thruster coefficients in the station-keeping phase.

Experiment	$u_{5}u_{6}\;RMS$	Depth RMSE (m)	Disturbance Thruster Coefficient	Thrusters’ Saturation
Control (C-T)	0.1275	0.011	0.00	No
1	0.1609	0.011	0.15	No
2	0.1720	0.013	0.25	No
3	0.1674	0.012	0.35	No
4	0.3546	0.024	0.50	No
5	0.5877	0.043	0.75	Yes
6	0.5810	0.040	1.00	Yes
7	0.1591	0.010	−0.15	No
8	0.1627	0.013	−0.25	No
9	0.2397	0.027	−0.35	No
10	0.2249	0.020	−0.50	No
11	0.2518	0.017	−0.75	No
12	0.3106	0.021	−1.00	No
13	0.1453	0.009	0.15 to 0.25	No
14	0.1275	0.009	0.15 to 0.25	No
15	0.1698	0.012	−0.15 to −0.25	No
16	0.2081	0.018	−0.15 to −0.25	No
17	0.2683	0.020	−0.35 to + 0.35	No
18	0.2312	0.017	−0.35 to + 0.35	No

**Table 5:** Experimental results for the $\tau_{z}$ control signal in the station-keeping phase.

Experiment	$\tau_{z}\;\mathrm{RMS}$	% vs. C-T	Disturbance Thruster Coefficient	Thrusters’ Saturation
Control (C-T)	5.88	-	0.00	No
1	8.95	152%	0.15	No
2	10.97	187%	0.25	No
3	11.63	198%	0.35	No
4	28.36	482%	0.50	No
5	49.57	843%	0.75	Yes
6	50.18	853%	1.00	Yes
7	7.98	136%	−0.15	No
8	9.79	166%	−0.25	No
9	16.03	273%	−0.35	No
10	14.95	254%	−0.50	No
11	20.14	342%	−0.75	No
12	25.12	427%	−1.00	No
13	10.39	177%	0.15 to 0.25	No
14	8.81	150%	0.15 to 0.25	No
15	10.43	177%	−0.15 to −0.25	No
16	13.64	232%	−0.15 to −0.25	No
17	21.46	365%	−0.35 to +0.35	No
18	18.50	314%	−0.35 to +0.35	No

## Data Availability

Data sharing is not applicable to this article.

## Abbreviations

The following abbreviations are used in this manuscript:

AUV	Autonomous Underwater Vehicle
ESC	Electronic Speed Controllers
PWM	Pulse Width Modulation
RMSE	Root Mean Square Error
RPi	Raspberry Pi
ROS	Robot Operating System
ROV	Remotely Operated Vehicle
SMC	Sliding Mode Control
SNAME	Society of Naval Architects and Marine Engineers
TBG	Time Base Generator
